# Invasive properties of patient-derived glioblastoma cells after reversible electroporation *in vitro*

**DOI:** 10.2478/raon-2025-0058

**Published:** 2025-12-16

**Authors:** Anja Blazic, Bernarda Majc, Metka Novak, Barbara Breznik, Lea Rems

**Affiliations:** 1 Faculty of Electrical Engineering, University of Ljubljana, Ljubljana, Slovenia; 2 Department of Genetic Toxicology and Cancer Biology, National Institute of Biology, Ljubljana, Slovenia; 3 Biotechnical Faculty, University of Ljubljana, Ljubljana, Slovenia; 4 Faculty of Chemistry and Chemical Engineering, University of Ljubljana, Ljubljana, Slovenia

**Keywords:** electroporation, high-frequency electric pulses, glioblastoma, patient-derived cells, invasion

## Abstract

**Background:**

Electroporation-based therapies are being explored in glioblastoma (GB) treatment, as means of enhancing drug delivery or achieving nonthermal ablation. Yet, little is known about how sublethal exposure affects the invasive behaviour of GB tumour cells.

**Materials and methods:**

Five patient-derived GB cell lines were initially screened for intrinsic invasive potential, and two most invasive (NIB140 CORE and NIB216 CORE) were selected for further experiments with electroporation treatment. Cells in suspension were exposed to bursts of high-frequency biphasic electric pulses resulting in electric field strength of 1 kV/cm, which corresponded to conditions of reversible electroporation. Changes in cell invasion and gene regulation were assessed 24 hours after electroporation using transwell assay and RNA transcriptome analysis, respectively.

**Results:**

Reversible electroporation at 1.0 kV/cm enhanced invasion in a cell line-dependent manner. NIB140 CORE showed a consistent and pronounced increase, with a median of 3.74-fold (274%) higher number of invading cells compared to sham control. In contrast, NIB216 CORE exhibited only a modest increase in invasion (1.30-fold; 30%). Transcriptomic profiling identified modulation of genes linked to extracellular matrix organization and ion channel activity in NIB140 CORE, and cytoskeletal remodelling in NIB216 CORE, indicating the activation of invasion-related pathways.

**Conclusions:**

These findings highlight a potential risk of pro-invasive responses in GB cells. In tumour ablation with irreversible electroporation, this concern relates to cells in the peripheral zone that may experience only sublethal electric fields, while in electrochemotherapy, a similar risk may arise if permeabilized cells are not effectively eliminated due to insufficient local drug delivery. Nevertheless, the two tested cell lines responded differently, underscoring patient-specific heterogeneity and the need for validation in more physiologically relevant models.

## Introduction

Electroporation is achieved by brief exposure of cells to high-intensity pulsed electric fields, creating nanoscale defects (i.e., pores) in the cell membrane. Depending on the extent of membrane disruption, cells may either restore homeostasis and survive (reversible electroporation) or fail to recover, leading to cell death (irreversible electroporation, IRE).^1^ Clinically, electroporation has gained recognition as a versatile tool in oncology. IRE can be used as a stand-alone, minimally invasive, non-thermal ablation technique^2,3^, whereas reversible electroporation can be used to enhance the uptake and cytotoxicity of chemotherapeutic drugs while allowing for reduced drug dosages (electrochemotherapy; ECT).^4,5^ Unlike thermal ablation, electroporation spares major blood vessels and the extracellular matrix, making it especially well-suited for tumours situated near vital or functionally critical structures.^2^ Moreover, by enhancing drug delivery and promoting anti-tumour immune activation, electroporation has become recognized as a key component of multimodal cancer therapy.^2^

Glioblastoma (GB), a WHO grade IV astrocytoma, is the most lethal and treatment-resistant primary brain tumour, with a median patient survival of around 15 months and a five-year survival rate below 10%.^6–8^ It is characterized by pronounced cellular and molecular heterogeneity, aggressive infiltration into surrounding brain tissue, and the development of a highly immunosuppressive microenvironment. Together, these biological features present significant challenges to developing effective treatments. The blood-brain barrier further limits drug delivery, while therapy-resistant GB stem cells and extensive genomic instability drive inevitable recurrence.^9^ Despite the fact that surgery, radiotherapy, and chemotherapy remain the standard treatments for GB, emerging evidence indicates that tumour cells surviving these treatments may acquire an even more invasive phenotype, further complicating disease management.^10^ This emphasizes the urgent need for novel, multimodal strategies capable of addressing complex tumour biology and preventing treatment-induced adaptation.

Given these challenges, there is growing interest in exploring alternative strategies for GB treatment. Several animal studies have demonstrated clinical potential of electroporation-based treatments for brain tumours. In canine models, research has primarily focused on IRE as a non-thermal ablation method. First-generation IRE protocols consisted of ninety 50-μs-long monophasic pulses at 4 Hz, producing well-controlled ablation volumes with sharp submillimeter transition zones between treated and healthy tissue.^11–13^ A notable prospective study using the NanoKnife system in seven dogs with spontaneous gliomas demonstrated safety and feasibility of IRE for brain tumour treatment.^14^ Individualized treatment plans were developed based on magnetic resonance image segmentation and computational optimization to ensure adequate electric field coverage of tumour by a sufficiently high electric field. Procedures involved craniotomy and stereotactic pulse delivery under general anaesthesia. Most adverse effects were mild to moderate and resolved with minimal intervention; however, two dogs experienced severe toxicity – one unrelated to IRE, and the other linked to the highest energy dose. Objective response was observed in four of five dogs with measurable lesions, with one dog remaining tumour-free for over five years.^15^ To address limitations such as muscle contractions and neuromuscular stimulation, second-generation high-frequency IRE (H-FIRE) protocols have been developed to minimize these undesired effects.^16^ A pilot study in three dogs with spontaneous meningiomas confirmed effective tumour ablation near critical vasculature with no major IRE-related side effects.^17^ In addition, the potential of ECT for GB treatment was demonstrated in rodent studies. In rats with induced gliomas, ECT with intravenous bleomycin improved their survival^18^, while intratumoral bleomycin combined with a newly designed electrode achieved complete tumour elimination in 69% of treated animals.^19^ Another study combining IRE and ECT with intravenous cisplatin via monopolar electrode showed delayed tumour growth and improved survival in gliomabearing rats.^20^ These results led to a phase I clinical trial (NCT01322100) investigating ECT for brain metastases, which was however discontinued due to low patient enrolment.^21^

Despite these encouraging findings, electroporation has not yet been clinically established for brain tumours. Treatment responses in preclinical studies were variable, and complete tumour control was not achieved in all animals. The underlying causes of this heterogeneity remain unclear. One contributing factor may be the inhomogeneous electric field distribution during treatment, which creates a central region of IRE surrounded by a narrow peripheral zone of reversibly electroporated cells.^22–24^ In highly infiltrative tumours like GB, some tumour cells are likely to be exposed only to sublethal electric field strengths, i.e. reversible electroporation, and survive the treatment. If electroporation alters the behaviour of surviving tumour cells, making them more invasive or aggressive, this might pose a potential risk for recurrence. A similar concern may arise in ECT, if insufficient drug delivery allows electroporated cells to survive the treatment. Thus, there is need for a deeper understanding of how reversible electroporation affects GB cells behaviour. Additionally, further preclinical studies are warranted, as even the most relevant animal models, such as spontaneous canine gliomas, still show important discrepancies compared to human GB. While animal gliomas can mimic human GB tumour heterogeneity and histological features, they include a lower number of mutated genes and a different immune cell response.^25–27^ Moreover, investigating the invasive behaviour of cells within sublethal regions is ethically and experimentally challenging *in vivo*, which further highlights the importance of clinically relevant *in vitro* models before progressing towards clinical application.

To investigate electroporation-induced changes in GB cell behaviour under clinically relevant conditions, we employed patient-derived primary cultures that more accurately reflect the genetic background, heterogeneity and invasive properties of human tumours compared to commercially available cell lines.^28^ This study was motivated by increasing evidence that sublethal therapies may promote a more aggressive phenotype in surviving tumour cells.^10,29^ Furthermore, our previous study^30^ revealed that reversible electroporation activates Ca^2+^-activated potassium channels in U-87 MG GB cell line, which are known to play a key role in regulating GB invasion.^31,32^ Therefore, we focused specifically on evaluating how electroporation affects the invasion of GB cells. We began by characterizing the invasive potential of five patient-derived GB cell lines and selected two cultures with the highest invasive capacities for further investigation. We then evaluated changes in tumour cell invasion induced by reversible electroporation. To ensure that we specifically examined the response of reversibly electroporated cells only, we employed a suspension-based approach, which provides a controlled system without the confounding effects of mixed reversible and irreversible populations. To gain deeper insight into how electroporation affects gene expression in surviving tumour cells, we additionally performed RNA sequencing in treated and non-treated samples. The findings presented here provide important insights that may contribute to the development of effective electroporation-based strategies for GB therapy.

## Materials and methods

### Cells

Experiments were performed using five different cell lines obtained from Slovenian GlioBank managed by the National Institute of Biology (NIB).^33^ Patients or their authorized representatives signed an informed consent in accordance with the Declaration of Helsinki. Collection and processing of tumour tissue material was approved by the National Medical Ethics Committee of the Republic of Slovenia (numbers 92/06/12, 0120-190/2018-4, 0120-190/2018-26, 0120-190/2018-32, and 0120-190/2018-35). Cell lines established from tumours were labelled with internal code numbers: NIB140 CORE, NIB216 CORE, NIB220 RIM, NIB237 CORE and NIB261 REC. CORE and RIM indicate the anatomical tumour regions from which the tumour cells were derived (the tumour core and infiltrative rim, respectively), while REC refers to cells isolated from a recurrent GB lesion. All cell lines were grown in Dulbecco’s Modified Eagle Medium (DMEM; Gibco, #41965039), supplemented with 10% foetal bovine serum (Gibco, #10500064) and antibiotics Penicillin-Streptomycin (Sigma-Aldrich, Germany, #P0781), hereafter referred to as DMEM10.

Cells were routinely passaged every 3 to 4 days and were maintained in a humidified environment at 37°C with 5% CO_2_. For determining the cell doubling time, 2x10^5^ cells were seeded per well of a 6-well plate (TPP, Switzerland), incubated at 37°C, 5% CO_2_, and then trypsinized and counted at selected times 20–100 hours after seeding. For electroporation, cells were trypsinized, counted, and centrifuged at 300 × g for 3 minutes. The resulting pellet was resuspended in DMEM10 with 10 mM HEPES, Sigma-Aldrich, #H0887 (hereafter referred to as DMEM10+) to achieve a final cell density of 1 × 10^6^ cells/ml.

### Electric pulse exposure

Cells were exposed to H-FIRE pulses, which were previously used in GB investigations *in vitro*,^34^ as well as *in vivo* for the treatment of spontaneous canine meningiomas^17^ and in a study examining blood-brain barrier disruption mechanisms.^35^ Specifically, we applied 100 bursts of biphasic pulses, with 2 μs negative and 2 μs positive phase, 5 μs interphase and 5 μs interpulse delay, 25 pulses/burst, at 1 Hz burst repetition frequency (Supplementary Figure S1). The pulse amplitude was varied between 100–400 V, corresponding to 0.5–2 kV/cm. Pulses were delivered by a high-frequency pulse generator L-POR (mPOR, Slovenia), through 2 mm electroporation cuvettes (VWR, #732-1136). The current and voltage were routinely monitored on an oscilloscope Wavesurfer 422, 200 MHz, using high-voltage differential probe ADP305 and current probe CP030 (all from Teledyne LeCroy, USA). The electric field to which the cells were exposed was estimated as the ratio between the applied voltage and the interelectrode distance.

We aimed to perform experiments at close-to-physiological temperature, which is relevant to *in vivo* tumour treatment. Thus, each cuvette was first preheated in an incubator at 33°C for at least 15 minutes. Subsequently, the cell suspension was added to the preheated cuvette, and placed back into the incubator at 33°C. Following an additional 10-minute incubation period, electric pulses were delivered to the cuvette inside the incubator. The temperature of 33°C was chosen based on our previous findings in U-87 MG GB cells, where electroporation at this temperature, but not at room temperature (~25°C), triggered activation of Ca^2+^-activated potassium channels that are associated with membrane hyperpolarization and increased invasive potential.^30^ In addition, responses at 33°C are expected to more closely approximate those at physiological temperature (37°C) than at room temperature, while maintaining a margin of safety against heating, as the sample temperature increased by > 8°C when the strongest electric pulses were delivered.

Joule heating of the sample due to pulse delivery was measured using a fibre optic sensor MPK-5 (OpSens Solutions, Canada). The sample temperature increased by 1.3°C ± 0.3°C at 200 V (1 kV/cm) and 8.3°C ± 0.7°C at 400 V (2 kV/cm), recordings shown in Supplementary Figure S2. This temperature increase was measured at room temperature (24–26°C); the increase during pulse delivery at 33°C is expected to be somewhat higher due to lower heat dissipation in warmer atmosphere.

### Permeabilization assay

Cell suspension (150 μl, 1 × 10^6^ cells/ml) prepared in DMEM10+ was mixed with propidium iodide (PI, Molecular probes, #P1304MP) in a final concentration of 100 μg/ml. PI is a nucleic acid stain that selectively penetrates cells with compromised membranes, where it binds to DNA and emits fluorescence. When added to cell suspension before pulse delivery, it enables identification of electroporated cells.^36^ 3 minutes after pulse application, 350 μl of electroporation solution was added to the cell suspension and the sample was removed from the electroporation cuvette. The percentage of PI-stained cells was quantified by flow cytometer (Attune NxT, Carlsbad, CA, USA) using blue laser excitation at 488 nm and detecting the emitted fluorescence through a 574/26 nm band-pass filter. 10,000 events representing individual cells were obtained, and data were analysed using the Attune Nxt software. Cells with fluorescence intensity above a certain gate value, defined based on fluorescence intensity histogram, were considered electroporated. Gating was set according to sham control (0 V). Measurements for each data point were repeated at least three times on three different days.

### PI-based viability assay

Cell suspension (150 μL, 1 × 10^6^ cells/ml) was prepared in DMEM10+ and transferred to an electroporation cuvette. After pulse application and additional 10-minute incubation at 33 °C, 850 μL of DMEM10+ was added to the cuvette. Afterwards, 100 μL of the treated cell suspension was plated into 24-well plate (TPP, Switzerland) containing 1 mL of DMEM10, and the plate was incubated at 37°C in a humidified atmosphere with 5% CO_2_ for 24 hours. PI was used to assess cell viability 24 hours after the electric pulse exposure. First, cells were harvested (attached and unattached) and centrifuged at 300 × g for 3 minutes. The cell pellet was then resuspended in 150 μL of growth medium together with PI in a final concentration of 100 μg/ml, and cells were incubated at room temperature for 5 minutes. The number of all cells (*N*_total_) and the number of PI-stained cells (*N*_PI+_) in a fixed sample volume was quantified by flow cytometer (Attune NxT; Life Technologies, USA), using a 488 nm blue laser and 574/26 nm band-pass filter. The percentage of viable cells was determined from (*N*_total_–*N*_PI+_)/*N*_total, ctrl_, as described in our previously published protocol^37^, where *N*_total, ctrl_ represents the total number of cells in sham control.

### MTS-based viability assay

Cells were prepared and exposed to electric pulses in the same way as for the Pl–based viability assay. 50 μL of the treated cell suspension was then plated into 96-well plate (TPP) containing 50 μL of DMEM10 and the plate was incubated at 37°C and 5% CO_2_. MTS metabolic assay (CellTiter 96 AQueous One Solution Cell Proliferation Assay, Promega, USA) was used to assess cell viability 24 hours after pulse exposure. Viable cells reduce the MTS tetrazolium compound into a soluble formazan product, the concentration of which correlates with the number of metabolically active cells and is determined by absorbance measurement. According to the manufacturer’s instructions, 20 μL of MTS tetrazolium compound was added to the samples, and the 96-well plate was returned to the incubator for 2 hours. The absorbance of formazan was measured with a plate reader (Tecan Infinite M200, Tecan, Austria) at 490 nm. The percentage of viable cells was calculated by subtracting the background (absorbance in wells with medium only) and normalizing the sample absorbance to the absorbance of the sham control.

### Transwell invasion assay

**FIGURE 1.**
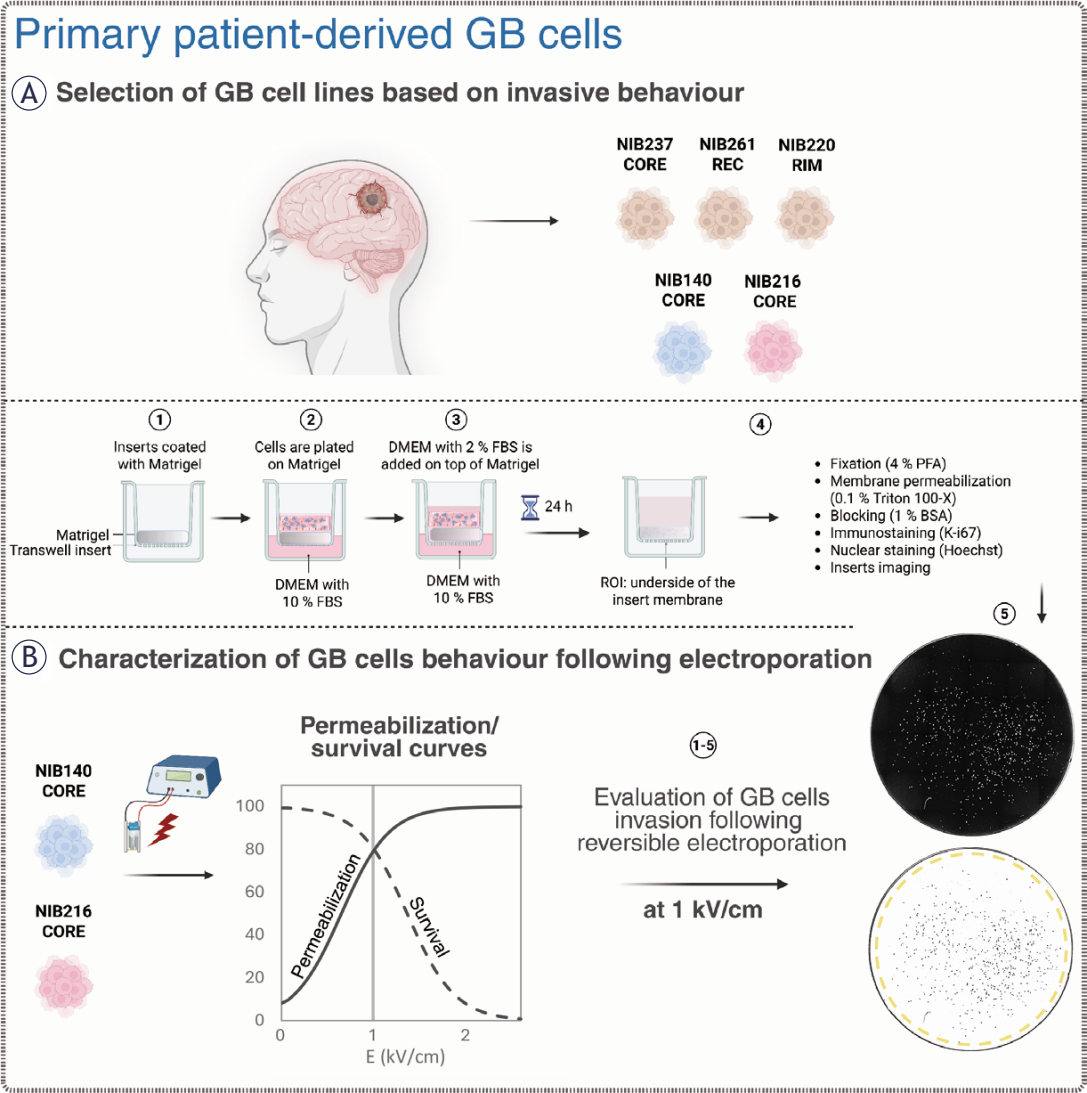
Overview of the experimental workflow for evaluating patient-derived glioblastoma (GB) cell behaviour before and after electroporation. Created with BioRender.com. **(A)** Five patient-derived glioblastoma cell lines, including cells from the tumour core (CORE), infiltrative rim (RIM), and a recurrent lesion (REC), were initially screened using a transwell invasion assay. Cells were plated on Matrigel-coated inserts and incubated for 24 hours. Invading cells migrating to the lower surface of the insert membrane were fixed, permeabilized, and stained with Hoechst (nuclei) and then immunostained for Ki-67 (a proliferation marker). The cells were subsequently imaged to quantify the number of invading and proliferating cells. **(B)** NIB140 CORE and NIB216 CORE were selected for further experiments with electroporation based on their invasive behaviour. Electric pulses of increasing electric field strength were applied to cells in electroporation cuvettes and the resulting membrane permeabilization and survival were quantified to generate characteristic response curves. Additionally, we assessed the metabolic activity of cells using MTS. Post-treatment invasion assay and fluorescence imaging was used to assess changes in invasive potential, with image analysis performed in ImageJ Fiji to quantify total and proliferating cell numbers based on nuclear segmentation and Ki-67 expression.

Transwell invasion assay was performed following a previously published protocol^38^, as shown in Figure 1. Transwell inserts containing membranes with 8.0-μm pores (Corning Life Sciences, #353097), pre-coated with Matrigel (Corning, #354234), were used to assess the invasive potential of the cell lines. A total of 25 μL of Matrigel solution, diluted 1:3 in DMEM supplemented with 2% FBS, was added to each insert and incubated at 37°C for 30 minutes to allow gelling. The lower chambers of 24-well plates were filled with 500 μL of DMEM10. To prevent premature polymerization, Matrigel was handled on ice using pre-cooled pipette tips throughout the procedure. For each insert, 80 000 cells (pre-treated with pulse exposure or not) were suspended in 100 μL of DMEM with 2% FBS and mixed with 50 μL of Matrigel diluted in DMEM to achieve a final Matrigel concentration of 0.5 mg/mL. After a 10-minute incubation at 37°C in a humidified 5% CO_2_ atmosphere, an additional 50 μL of DMEM with 2% FBS was added to each insert, resulting in a final volume of 200 μL. The inserts were then incubated for 24 hours.

Following incubation, non-invading cells and remaining Matrigel were removed from the upper surface of the membrane using a cotton swab. The inserts were transferred to fresh wells containing 500 μL of DPBS (Gibco, #14190) and washed twice. Cells on the underside of the membrane were fixed with 4% paraformaldehyde (Sigma-Aldrich, # 158127) for 15 minutes at room temperature, followed by two DPBS washes. Membranes were then incubated in DPBS containing 1% bovine serum albumin (BSA; Sigma-Aldrich, #A2153 or Fisher BioReagents, #BP9702) and 0.1% Triton X-100 (Sigma-Aldrich, #T8787) for 30 minutes at room temperature to block non-specific binding. For proliferation assessment, Ki-67 FITC-conjugated antibody (Miltenyi Biotec, #130-117-691) was added at a 1:50 dilution in DPBS, and membranes were incubated for 1 hour at room temperature. After one PBS wash, cell nuclei were counterstained with Hoechst 33342 (Thermo Fisher Scientific, #62249) diluted 1:1000 in PBS and incubated for at least 5 minutes.

Transwell invasion and proliferation assays were performed in five GB cell lines (NIB 140 CORE, NIB216 CORE, NIB220 RIM, NIB237 CORE and NIB261 REC) and selected electroporated samples (NIB 140 CORE and NIB216 CORE) to evaluate treatment-induced changes in GB cell behaviour. Tile-scan imaging of the entire membrane undersurface with invading cells was carried out using two fluorescence microscopy systems. For characterizing baseline invasion in all five GB cell lines, cells were imaged using the EVOS FL Auto 7000 system (Thermo Fisher Scientific, USA), which employed both brightfield and fluorescence channels to visualize nuclei stained with Hoechst and proliferating cells labelled with Ki-67 under 10× objective magnification. Imaging was performed using excitation wavelengths of 395 nm for Hoechst and 475 nm for Ki-67. For characterizing invasion in electroporated cells and corresponding sham control groups, the same fluorescence channels were used to image the samples on the Leica Thunder Imaging System with DMi8 inverted epifluorescence microscope and LED8 illumination source controlled by Las X software (all from Leica Microsystems, Germany) under 10× objective magnification.

Image analysis was performed using ImageJ Fiji.^39^ Nuclei were first segmented based on Hoechst staining (as presented in Figure 1), and the resulting regions of interest (ROIs) were applied to the Ki-67 channel to extract signal intensity and determine proliferation status. Quantification of invading and proliferating cells was performed across at least three independent experiments.

### Statistical analysis

All results are presented as mean ± standard deviation (SD), based on a least of three independent experiments performed on separate days. Statistical analyses were conducted using SigmaPlot version 11.0 (Systat Software Inc., San Jose, CA, USA), with analyses performed separately for each cell line. Normality was assessed using the Shapiro–Wilk test, and homogeneity of variance was evaluated using Levene’s test. For datasets meeting assumptions of normality and equal variance, one-way ANOVA was applied, followed by Holm–Sidak’s post hoc test for multiple comparisons. When assumptions were not met, nonparametric ANOVA on ranks was used, followed by Dunn’s post hoc test. For comparisons involving two groups only, a Student’s t-test was used when normality and variance assumptions were satisfied; otherwise, a Mann-Whitney U test was applied. A p-value < 0.05 was considered statistically significant.

### RNA transcriptome analysis

Total RNA was extracted from GB cells (NIB140 CORE and NIB216 CORE) using the E.Z.N.A.® Total RNA Kit I (Omega Bio-Tek, Norcross, GA, USA; Cat. No. R6834). To replicate the conditions used in the Transwell invasion assay, cells were first exposed to an external electric field as described in the section above. Ten minutes following pulse exposure, 850 μL of DMEM10+ was added directly to the electroporation cuvette. The full volume was then transferred to a single well in 6-well plate, and an additional 2 mL of DMEM10 was added, bringing the total volume per well to 3 mL. Sham-treated control cells were handled identically but were not subjected to pulse exposure. The total RNA was extracted 24 hours after the pulse exposure.

Transcriptome analysis was performed by NovoGene (Munich, Germany). Total RNA was extracted from electroporated and sham control samples and subjected to quality control using the RNA Nano 6000 Assay Kit of the Bioanalyzer 2100 system (Agilent Technologies, CA, USA). mRNA was purified from total RNA using poly-T oligoattached magnetic beads, fragmented, and reverse transcribed into cDNA. After second-strand synthesis and adaptor ligation, libraries containing 370–420 bp fragments were purified using the AMPure XP system and subsequently amplified by PCR. Following amplification, PCR products were purified again. Library quality was assessed using the Agilent Bioanalyzer 2100, and clustering was performed on a cBot Cluster Generation System using the TruSeq PE Cluster Kit v3-cBot-HS (CA, USA). The libraries were then sequenced on an Illumina NovaSeq platform, generating 150 bp paired-end reads. Raw reads were processed using fastp for adapter trimming and quality filtering. Clean reads were aligned to the reference genome using HISAT2 (v2.0.5), and transcript assembly was performed with StringTie (v1.3.3b). Gene-level read counts were generated with featureCounts (v1.5.0-p3), and gene expression was quantified as fragments per kilobase of transcript per million mapped reads (FPKM), which accounts for both transcript length and sequencing depth.

Differential gene expression analysis was performed in NovoMagic (https://eu-magic.novogene.com/) using DESeq2 (v1.20.0), based on a negative binomial model. Gene ontology (GO) enrichment analysis was conducted using the clusterProfiler R package, correcting for gene length bias. GO terms with adjusted p < 0.05 were considered significantly enriched. For visualization, unadjusted p-values (p ≤ 0.05) were used in volcano plots to highlight global transcriptional changes, whereas adjusted p-values (Benjamini-Hochberg correction) were used in GO enrichment plots to account for multiple testing and reduce false discovery.

The raw RNA-seq data are publicly available in the Gene Expression Omnibus (GEO) repository under accession number GSE305017.

## Results

### Selection of patient-derived GB cell lines based on their invasive properties

**FIGURE 2.**
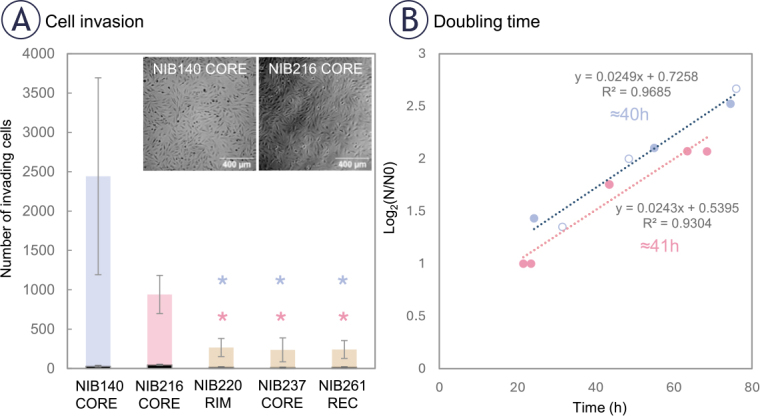
Patient-derived glioblastoma (GB) cell lines display variable intrinsic invasive potential. **(A)** Transwell invasion assay was performed with non-treated cell lines to assess the intrinsic invasive potential of five GB cell lines derived from different tumour regions. NIB140 CORE showed the highest number of invading cells, followed by NIB216 CORE, whereas NIB220 RIM, NIB237 CORE, and NIB261 REC displayed significantly lower invasion. Statistical analysis was performed using ANOVA on ranks. Significant differences are indicated with asterisks (*); p < 0.05. The number of Ki-67 positive (proliferating) cells, shown in black at the base of each bar, was low in all tested cell lines (< 10 %). Data are presented as mean ± SD from at least 4-5 independent experiments. **(B)** Doubling times were determined based on cell growth curves plotted as log_2_(N/N_0_) versus time, where N_0_ is the number of seeded cells at time 0 h, and N is the number of cells at selected time points (hours). Linear regression was applied to each cell line (R^2^ values shown), and doubling time was calculated from the slope of the fitted line. NIB140 CORE and NIB216 CORE showed similar doubling time (40–41 h).

To characterize heterogeneity in invasive behaviour among patient-derived GB cell lines, we performed a standardized transwell invasion assay (Figure 1) using five lines representing distinct tumour regions. As shown in Figure 2, invasive potential varied markedly across the cell lines. NIB140 CORE and NIB216 CORE exhibited the highest levels of invasion, while NIB220 RIM, NIB237 CORE, and NIB261 REC displayed significantly lower invasive activity compared to NIB140 CORE and NIB216 CORE (ANOVA on ranks, p < 0.05). Based on their invasion profiles, NIB140 CORE and NIB216 CORE were selected for subsequent experiments to investigate electroporation responses across the two GB subtypes representing the highest levels of invasion. After 24 hours, the expression of the proliferation marker Ki-67 was low in all tested cell lines (< 10%), confirming that the observed invasion was not driven by cell proliferation, as shown in Figure 2A. The number of proliferating cells is represented at the base of each bar, illustrating that proliferation does not account for the observed invasive behaviour.

To further confirm that the observed invasion was not driven by proliferation, we measured the doubling time of each cell line. NIB140 CORE and NIB216 CORE displayed doubling times of ~40 and ~41 hours, respectively. Representative growth curves used for this estimation are shown in Figure 2B, illustrating that the 24-hour post-treatment time point falls well before either population is expected to divide. This supports the interpretation that the observed behaviour reflects actual invasion properties rather than proliferative expansion.

### Permeabilization and survival at different electric field strengths

**FIGURE 3.**
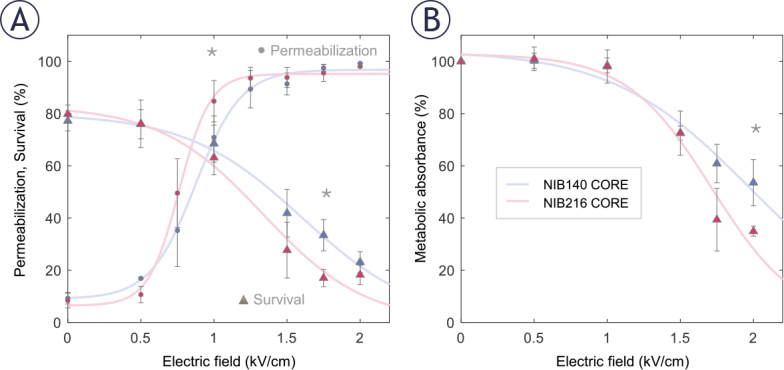
Permeabilization and survival of NIB140 CORE and NIB216 CORE glioblastoma (GB) cell lines in response to H-FIRE pulses resulting in different electric field strengths. **(A)** The percentage of permeabilized cells was assessed by propidium iodide (PI) uptake 3 minutes after pulse delivery (presented as •). The percentage of viable cells was assessed by PI assay 24 hours after pulse delivery (presented as ▴). **(B)** Cell survival was assessed by metabolic MTS assay 24 hours after pulse delivery. Data are presented as mean ± SD from at least three independent experiments. Solid lines are least-square fits to sigmoid curves. Statistically significant differences (p < 0.05) between cell lines at specific electric field strengths were tested using Student’s t-test and are indicated by asterisks (*). Data for NIB140 CORE and NIB216 CORE are shown in blue and pink, respectively.

We next investigated how the selected NIB140 CORE and NIB216 CORE cell lines respond to pulses of increasing electric field intensities. Membrane permeabilization was assessed 3 minutes after electroporation using propidium iodide (PI) staining, while cell survival was evaluated 24 hours post-treatment using both PI staining and the metabolic MTS assay. Both NIB140 CORE and NIB216 CORE exhibited a characteristic sigmoidal increase in the percentage of permeabilized cells with increasing electric field strength, reaching maximal values above 1.25 kV/cm (Figure 3A). Survival determined by PI assay declined above 1 kV/cm (Figure 3A). These results align with previous H-FIRE studies demonstrating that glioma cells can recover metabolic activity and proliferative capacity when exposed to sublethal electric fields, whereas higher intensities induce irreversible membrane damage.^34^

NIB216 CORE displayed somewhat greater permeabilization at intermediate electric field strength and a more pronounced decrease in viability at higher field strengths compared to NIB140 CORE, indicating greater sensitivity to electroporation-induced stress. This was further supported by MTS assay results (Figure 3B), which showed a greater reduction in metabolic activity in NIB216 CORE. Statistically significant differences (Student’s t-test) between the two cell lines were observed at 1 kV/cm for membrane permeabilization (p=0.037), 1.75 kV/cm for survival (p=0.001), and 2 kV/cm for metabolic activity (p=0.024), with significant differences indicated by asterisks (Figure 3). Nevertheless, the differences between the tested cell lines were relatively small, suggesting that similar electric field strengths can be used to treat different GB cell lines.

### Reversible electroporation enhances invasion of GB cells in a cell typedependent manner

Based on permeabilization and survival curves (Figure 3), we chose an electric field strength of 1.0 kV/cm to further assess whether sublethal electroporation alters GB cell invasion. At this electric field strength, both NIB140 CORE and NIB216 CORE cell lines reached > 80% permeabilization while maintaining viability above 80% relative to sham-treated control (0 kV/cm). The chosen electric field strength mimics the conditions in reversibly electroporated border zone surrounding the ablated area, when using IRE for tumour treatment. Also, these conditions of reversible electroporation are in line with clinically relevant protocols used for ECT.^40^

**FIGURE 4.**
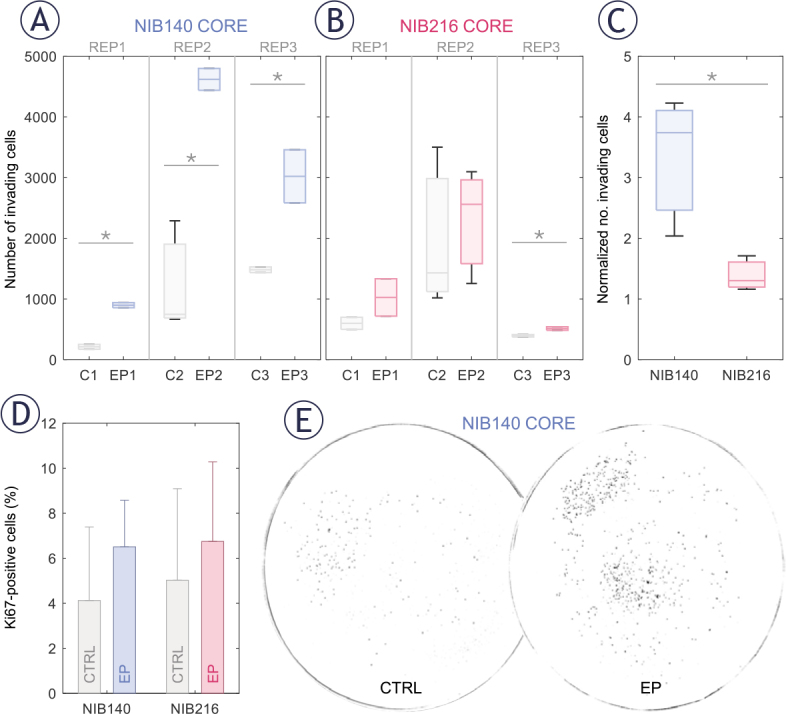
Electroporation enhances the invasion potential of patient-derived glioblastoma (GB) cell lines in a cell type-dependent manner. Invasion was assessed 24 hours after electroporation using H-FIRE pulses resulting in electric field strength of 1 kV/cm. **(A-B)** Box-and-whisker plots showing the number of invading cells in NIB140 CORE **(A)** and NIB216 CORE **(B)** in sham-treated (grey) and electroporated samples (blue or pink). Each group represents a separate biological replicate (REP1–REP3), with 2–3 technical replicates per biological replicate. The horizontal line within each box represents the median, and whiskers indicate the full range of values. **(C)** Relative increase in the number of invading cells in electroporated samples compared to sham controls. Data are presented as mean ± SD from three biological replicates. **(D)** Percentage of Ki-67–positive (proliferating) cells in sham-treated and electroporated samples. Values remained below 10 % across all conditions, demonstrating that the observed increase in invasion was not due to increased proliferation. **(E)** Representative masks obtained after thresholding images of Hoechst-stained NIB140 CORE invading cells, showing increased invasion following electroporation.

Electroporation enhanced the invasion potential of GB cells in a cell type-dependent manner, as quantified 24 hours following exposure to 1.0 kV/cm. Since the number of invading cells varied from day to day, already in control samples, we present results for each of the three biological replicates separately, with 2–3 technical replicates (transwell inserts) per one biological replicate. In NIB140 CORE, the number of invading cells was consistently and significantly higher in electroporated samples compared to sham-treated controls across all three biological replicates (Figure 4A; Student’s t-test, *p* < 0.05; 2–3 technical replicates per one biological replicate). In contrast, NIB216 CORE showed a more variable response, with significance reached in one biological replicate only (Figure 4B), indicating a modest and less consistent effect. We then averaged the technical replicates and normalized this averaged number of invading cells in electroporated samples to the corresponding number in sham-treated controls for each biological replicate. The obtained foldincrease in invading cells across biological replicates is presented in the box plot in Figure 4C. This analysis confirmed a consistent increase in invasion in NIB140 CORE and only modest trend in NIB216 CORE. Notably, NIB140 CORE exhibited a significantly greater 3.74-fold increase compared to just 1.30-fold in NIB216 CORE (Student’s t-test, *p* < 0.05), potentially reflecting intrinsic differences in these cell lines.

Enhanced cell invasion following sublethal electroporation was further supported by analysis of the proliferation marker Ki-67. The proportion of Ki-67–positive cells remained below 10% across all conditions (Figure 4D), with no significant differences between electroporated and sham-treated controls (Student’s t-test). These findings reinforce the conclusion that proliferation did not contribute considerably to the increased number of invading cells following electroporation. An example of this electroporation-induced increase in invasion potential in NIB140 CORE cell line is illustrated in Figure 4E, where representative images demonstrate a higher number of invading cells after electroporation at 1 kV/cm.

### RNA transcriptome analysis corroborates enhanced invasion of reversibly electroporated GB cells

**FIGURE 5.**
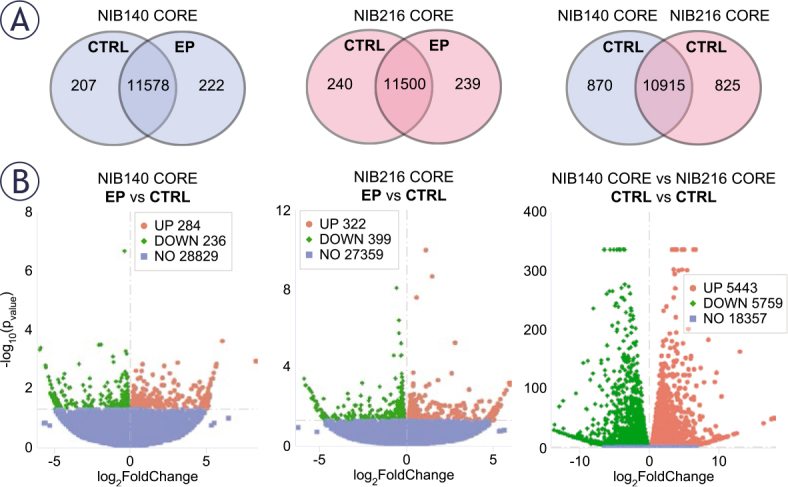
Transcriptomic differences between electroporated and shamtreated NIB140 CORE and NIB216 CORE cell lines. RNA transcriptome analysis was performed in cells harvested 24 hours after electroporation. **(A)** The gene expression levels analysis is presented through co-expression Venn diagrams showing the overlap in expressed genes between sham-treated (CTRL, 0 V) and electroporated (EP, 1 kV/cm) samples of each cell line, and between shamtreated NIB140 CORE and NIB216 CORE. **(B)** The differential gene expression analysis is presented through volcano plots. Red and green points represent significantly upregulated and downregulated genes, respectively (p < 0.05), while blue points indicate non-significant changes. Genes were classified as differentially expressed, if they met the threshold of |log_2_FoldChange| > 0.0.

To gain insight into the molecular changes associated with electroporation, we performed RNA sequencing on NIB140 CORE and NIB216 CORE cells harvested 24 hours after exposure to 1.0 kV/cm. Gene expression level analysis in electroporated (EP) and sham-treated (CTRL) samples, presented through co-expression Venn diagrams (Figure 5A), revealed 222 and 239 genes that were uniquely expressed in the electroporated NIB140 CORE and NIB216 CORE samples, respectively. Differential gene expression analysis, presented through volcano plots (Figure 5B) further confirmed electroporation-induced transcriptomic changes in the two cell lines, with both significantly downregulated and upregulated genes.

Additionally, comparison between the shamtreated NIB140 CORE and NIB216 CORE revealed that these cell lines considerably differ in their baseline transcriptomic profiles. Co-expression Venn diagram (Figure 5A) showed 10,915 genes coexpressed in both cell lines, with 870 and 825 genes uniquely expressed in NIB140 CORE and NIB216 CORE, respectively. Volcano plot (Figure 5B) further confirmed the large transcriptomic divergence between the two cell lines. This divergence indicates that the intrinsic transcriptomic differences between NIB140 CORE and NIB216 CORE exceed the shifts induced by electroporation, which may explain the different extents to which electroporation changed the invasion of these two cells lines (Figure 4).

**FIGURE 6.**
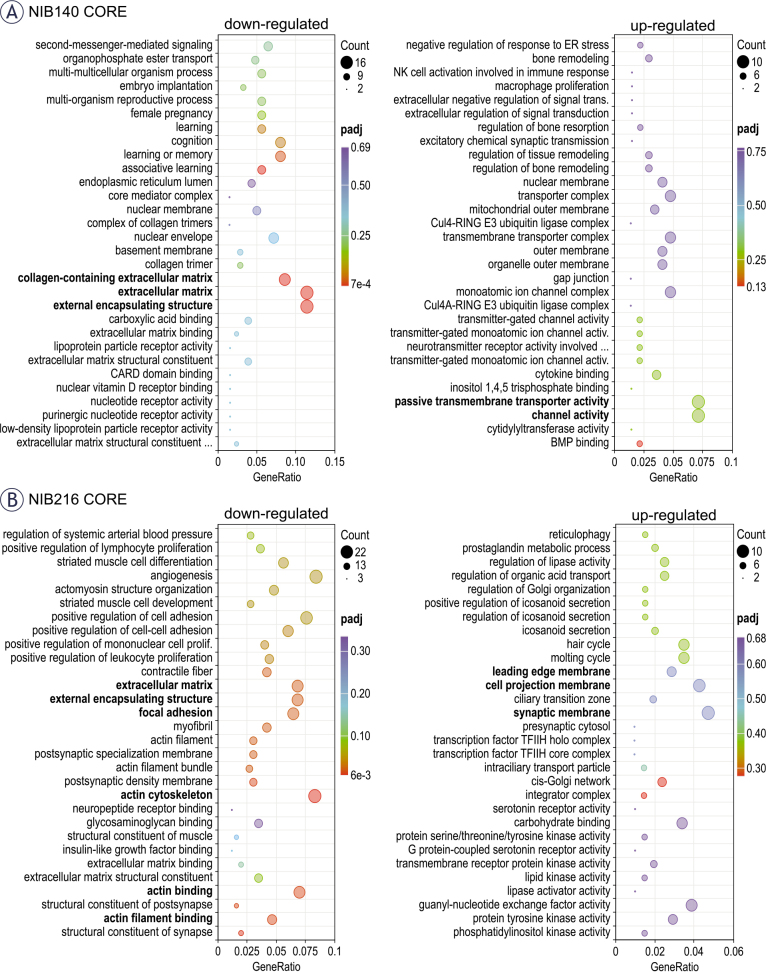
Functional enrichment analysis of differentially expressed genes following electroporation. **(A)** Gene ontology (GO) enrichment analysis of significantly upregulated (right) and downregulated (left) genes in NIB140 CORE cells 24 hours after electroporation. **(B)** GO enrichment analysis for NIB216 CORE. Dot size reflects the number of genes contributing to each GO term, while colour intensity indicates statistical significance (adjusted p-value, padj). The GeneRatio represents the proportion of differentially expressed genes associated with each GO term relative to the total number of input genes. Selected invasion-relevant categories are highlighted in bold. GO terms include biological processes, molecular functions, and cellular components.

To better understand the biological relevance of the observed transcriptomic changes, we performed gene ontology (GO) enrichment analysis on significantly upregulated and downregulated genes in NIB140 CORE and NIB216 CORE cells following electroporation (Figure 6). In NIB140 CORE, differentially expressed genes were enriched in invasion-associated pathways, including channel activity and extracellular matrix organization (Figure 6A), which aligned with the observed increase in invasion (Figures 4A, C). In contrast, the transcriptional response in NIB216 CORE lacked strong enrichment of motility-related pathways, consistent with the modest increase in invasion (Figures 4B, C). However, several downregulated categories in NIB216 CORE—including actin filament binding, actin cytoskeleton, extracellular matrix, and focal adhesion—suggest cytoskeletal remodelling and/or disruption. In addition, genes associated with leading-edge membrane, cell projection membrane, and synaptic membrane were upregulated. These differences underscore the intertumoral variability in molecular responses to electroporation and support a potential mechanistic link between transcriptomic changes and the significantly enhanced invasion observed in the NIB140 CORE cell line, which would, however, need to be further supported at the functional level.

## Discussion

Our study investigated how sublethal exposure to electroporation pulses affects the invasion of GB tumour cells. After initial screening of five patient-derived GB cells lines for their intrinsic invasive potential, we selected the two most invasive cell lines (NIB140 CORE and NIB216 CORE) for further electroporation experiments. We characterized cell permeabilization and survival after exposure to H-FIRE pulses resulting in different electric field strengths and found that 1 kV/cm corresponds to conditions of reversible electroporation in both cell lines. At 1 kV/cm, the majority of cells became permeabilized due to electroporation while still retaining their viability 24 hours later. We then assessed changes in their invasion behaviour 24 hours after electroporation. Electroporation enhanced invasion in a cell line–dependent manner: NIB140 CORE consistently showed a pronounced response with a median 3.74-fold higher number of invading cells compared to sham-treated controls. While the number of invading cells was consistently higher in electroporated samples, we observed a rather high variability across biological replicates. This variability can be explained by the use of patient-derived cells, which are expected to respond more heterogeneously than established cell lines that often fail to replicate key tumour characteristics.^28,33,41^ Unlike in NIB140 CORE, electroporation induced only a modest increase in the number of invading cells in NIB216 CORE (1.30-fold). Moreover, NGS-based profiling included in the clinical pathology report identified the *EGFRvIII* variant in NIB140 CORE cell line (but not in NIB216 CORE), a mutation known to enhance invasion and contribute to treatment resistance in GB.^42^

To better understand the molecular basis of increased invasion after electroporation and the associated differences between the two tested cell lines, we performed transcriptomic analysis. In NIB140 CORE cells, we observed upregulation of genes associated with the channel activity and passive transmembrane transport activity – *CHRNE, KCNMA1, KCNAB1, TRPC4, GJC3, GPR89A, TTYH2, GRIN2A, RHCE*, and *GLRA3*.^43^ Notably, ion channel–related genes such as *KCNMA1* and *KCNAB1*, i.e. the alpha and beta subunits of the big potassium K_Ca_ channel, were detected, supporting their potential role in enhanced invasive behaviour in GB observed in previous studies.^29,32^ Genes related to extracellular matrix (ECM) organization (collagen containing ECM, ECM and external encapsulating structure) were downregulated *(COL14A1, EFEMP1, ITGB4, COL8A1, P3H2, THBS2, INHBE, MATN4, PTPRZ1, MMP9, ANGPTL5* and *COL5A2)* indicating ECM remodelling.^44,45^ In this context, it is notable that MMP9, a metalloproteinase classically associated with invasion, was downregulated in NIB140 CORE. This may appear counterintuitive given the observed increase in invasion, but it is consistent with reports that GB cells can compensate protease activity by other protease families or proteases of the same family, adopt protease-independent, ion channel- and adhesion-driven or even adhesion-independent migration strategies.^46^ Thus, while MMP9 itself was not upregulated, ECM- and ion channel–related pathways were altered, supporting the idea that alternative mechanisms may drive invasion in this context.^46^ In contrast, NIB216 CORE showed downregulation of genes involved in cytoskeleton remodelling and focal adhesion *(COL11A1, CNN1, ALPL, HAPLN1, TGFB1I1, F3, IGFBP7, ADAM19, COL4A1, POSTN, LOXL4, MXRA7, CCN2, LGALS1, COL4A2, GPC4, TFPI2, CD248, VASP, TAGLN, TPM2, PDLIM7, PPP1R18, ARPC4, CORO1A, ACTN1, FHDC1, PICK1, SPTBN2, ADSS1, MYOZ1, TMEM201, MARCKSL1* and *MYH9)* suggesting cytoskeletal disruption.^47^ Meanwhile, upregulated response was linked to membrane dynamics – leading edge membrane, cell projection membrane and synaptic membrane (*ANK1, DPP4, LAMP5, EGFR, C2CD5* and *PSD3*) indicating changes in membrane plasticity and intracellular communication.^48^ It should be noted that, based on our data, we cannot determine whether the observed effects arise directly from pulse-induced biophysical changes or indirectly through stress-mediated signalling. Furthermore, this data should be interpreted with caution, as validation at the protein level (e.g., Western blot or ELISA) will be required to confirm whether the observed gene expression changes translate into functional effects.

A recent study by Wang *et al*.^49^ reported that electroporation suppresses invasion of U-87 MG GB cells. Similar to our study, cells in suspension were electroporated and changes in invasion were assessed 24 hours later using a transwell invasion assay. The pulse parameters used for electroporation was somewhat different from ours and consisted of 4–8 bursts of 50 biphasic 2 μs pulses with 0.2 μs interphase and 100 μs interpulse delay, 15 Hz burst repetition frequency, and 4 kV/cm electric field strength. With 6 and 8 bursts, cell survival dropped to ~73% and 42%, respectively, and this decrease in the number of viable cells was expectedly reflected in lower number of invading cells. Nevertheless, the number of invading cells decreased to ~56% of control also with 4 bursts, where ~90% cells survived the treatment. Decreased invasion was associated with downregulation of *SIRT1* gene and *SIRT2* genes and impaired mitochondrial function. In contrast, we observed increased invasion and no significant changes in any of *SIRT1–7* genes (p=0.1; Benjamini-Hochberg correction) in our study. Furthermore, we observed a trend of increased invasion even at higher electric field strength of 2 kV/cm, after compensating for the reduced number of surviving cells, although this increase in invasion was not statistically significantly different from control, results presented in Supplementary Figure S3. The different results obtained by us compared to Wang *et al*.^49^ could stem from multiple reasons. Aside from differences in pulse parameters and sample temperature during electroporation, we used patient-derived GB cells lines. As shown by our transcriptomic analysis, different GB cells lines have considerably different gene expression profiles, which affects their response to electroporation. This highlights the value of patient-derived models in capturing clinically relevant transcriptional responses and treatment dynamics compared to immortalized cell lines. The importance of using patient-derived cells to better capture the biological complexity and treatment responses of GB is further illustrated by comparing our results to the study by Casciati *et al*.^50^ In this study, adherent U-87 MG cells were exposed to five electric pulses, each lasting 40 μs at 1 Hz and 30 kV/cm (0.3 MV/m). While they also cultured neurospheres under serum-free conditions to enrich for GB stem-like cells, these were still derived from the U-87 MG line, which lacks key features of primary tumours, including heterogeneity and true invasive behaviour.^51^ Notably, Casciati *et al*. reported that pulse exposure substantially influenced the fate of GB neurospheres by differentially regulating genes involved in hypoxia, inflammation, and p53/cell cycle checkpoints, ultimately reducing their capacity for neurosphere formation and transmigration *in vitro*. Furthermore, pulse exposure also reduced the ability to form new neurospheres and inhibited invasion. Importantly, exclusively in U-87 neurospheres, pulse exposure altered the expression of stemness- and differentiation-related genes. While these findings are promising, the observed inconsistency with our results—despite differences in pulse parameters—might reflect cell modelspecific differences in electroporation responses. This highlights the need to validate such effects in more physiologically relevant models. Given the aggressive, therapy-resistant nature of GB stemlike cells and their contribution to tumour progression and recurrence^52,53^, future electroporation studies should consider the use of patient-derived stem-like populations to more accurately reflect clinically relevant outcomes.

While most preclinical studies of electroporation-based brain tumour therapy have focused on IRE as a non-thermal ablation method, particularly in canine glioma models^11,12,14,17,54^, our findings highlight the less-explored effects on tumour cells located in the periphery of IRE-treated zones. This raises an important consideration regarding unintended effects in tumour margins that remain viable after treatment—regions likely exposed to sublethal electric fields due to the highly infiltrative nature of GB. Our results demonstrate that tumour cells surviving electroporation may acquire enhanced invasive potential, a concern that arises specifically when no cytotoxic agents are present. However, a similar concern applies to ECT if insufficient local drug concentrations are achieved, since permeabilized cells might survive the treatment. When adequate concentrations are ensured, ECT directly addresses this risk by eliminating reversibly permeabilized cells through enhanced intracellular accumulation of cytotoxic agents, such as bleomycin and cisplatin.^18,19^ Bleomycin induces DNA strand breaks, while cisplatin causes DNA crosslinking and apoptosis—mechanisms that require cytosolic access and are otherwise ineffective across intact membranes.^55–56^ Since the primary effect of electroporation is to increase membrane permeability, it provides a unique opportunity to deliver these otherwise impermeable drugs efficiently. In addition, electroporation has been shown in *in vivo* models to transiently disrupt the blood–brain barrier, further highlighting its potential for enhancing drug delivery to tumour tissue within the central nervous system.^35,57,58^ Moreover, studies in melanoma cells showed that ECT does not affect the cells’ metastatic potential.^59^,^60^ Taken together, our findings suggest that ECT, by combining reversible electroporation with sufficient concentrations of cytotoxic agents, may offer a more effective and safer therapeutic strategy for glioblastoma than IRE as a standalone treatment. Furthermore, this approach may help overcome some limitations of current chemotherapy regimens, such as temozolomide, which has been shown to expand the GB stem cell population through conversion of differentiated tumour cells both *in vitro* and *in vivo*.^61^

While our results offer new insights into GB cell responses to electroporation, this study has several limitations. First, the use of suspension cultures does not fully recapitulate the structural complexity, cell–cell interactions, and diffusion gradients present *in vivo*. These factors may influence electroporation-induced processes such as membrane repair, intracellular signalling, and invasion. Although patient-derived GB cells were used, future studies should also examine cells from spatially distinct tumour regions (e.g., core vs. rim), which may exhibit different responses due to intratumoral heterogeneity. In addition, GB stem-like cells, known for their high invasion potential and therapy resistance^52,63^, were not specifically addressed here and represent a critical subpopulation for further investigation. To better approximate the tumour microenvironment, future experiments should employ advanced *in vitro* models such as multicellular spheroids or organoids, which incorporate three-dimensional architecture and preserve key features of GB biology, including heterogeneity, invasion, and treatment resistance. Arroyo *et al*.^63^ have recently advanced this field by developing a multicellular spheroid–hydrogel platform, demonstrating that higher electric field strengths and longer pulse widths constrained migration and proliferation over several days, underscoring the importance of 3D models for validating electroporation responses. Finally, this study focused on short-term transcriptional and behavioural changes, with analysis limited to the 24-hours timepoint following electroporation. Long-term effects were not addressed here and remain to be explored, particularly in the context of combination therapies. Experiments were performed at 33°C to build on prior findings of ion channel activation in GB cells^30^, while also minimizing the risk of thermal damage. Future studies could further examine temperature dependence alongside 3D models to better approximate physiological conditions. Moreover, future work should investigate how electroporation interacts with established treatments, including radiation and chemotherapeutic agents such as temozolomide, cisplatin, or bleomycin, to better understand the impact on cell viability and invasion.

Overall, our findings suggest that sublethal electroporation can enhance GB cell invasion potential in a cell line-dependent manner. A more pronounced and consistent effect was observed in NIB140 CORE cells (3.74-fold increase), while NIB216 CORE showed only a modest increase (1.30-fold) in the number of invading cells following reversible electroporation. While our findings suggest that combining reversible electroporation with sufficient concentrations of cytotoxic agents (ECT) may offer advantages over IRE alone, this requires further validation in more physiologically relevant models.

## Use of Al-assisted technologies in the writing process

During the preparation of this paper, the authors used Claude (Anthropic, CA, USA) and ChatGPT (OpenAI, CA, USA) to improve the style and readability in some parts of the text. After using this tool/service, the authors have reviewed and edited the content as required and take full responsibility for the content of the publication.

## Supplementary Material

Supplementary Material Details
