# Supplementary material for: Invasive properties of patient-derived glioblastoma cells after reversible electroporation in vitro
Source: Radiol Oncol. 2025 Dec 16;59(4):535–50. doi: 10.2478/raon-2025-0058 (PMC12707454; doi:10.2478/raon-2025-0058)
Supplement: Supplementary file 1 — Supplementary Material Details [file raon-2025-0058_sm.pdf]

Invasive properties of patient-derived glioblastoma cells after reversible electroporation *in vitro*

Anja Blazic, Bernarda Majc, Metka Novak, Barbara Breznik, Lea Rems

doi: 10.2478/raon-2025-0058

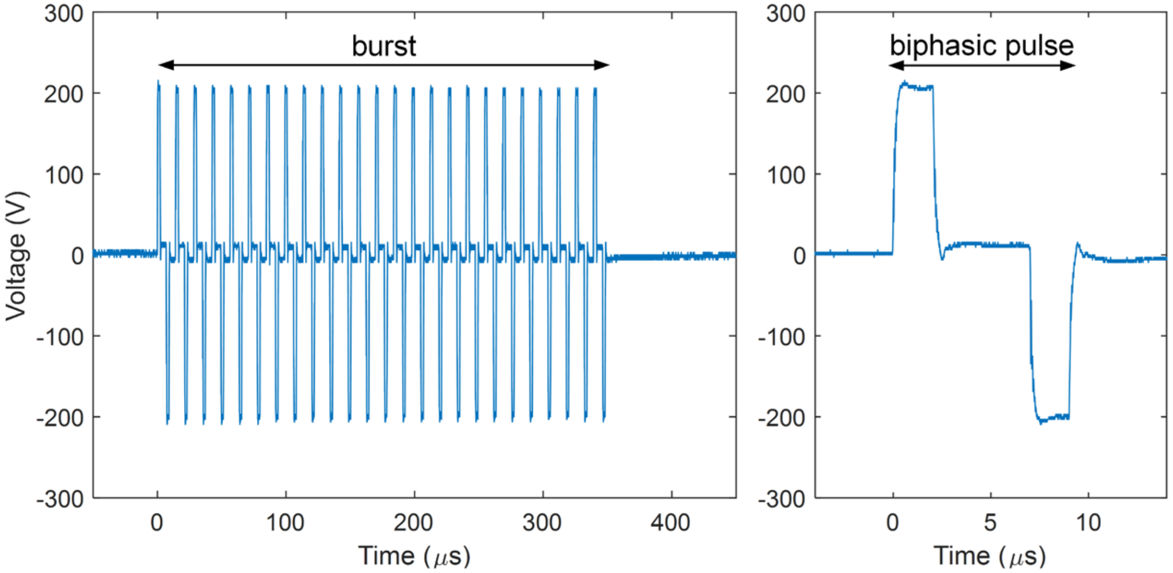

SUPPLEMENTARY FIGURE S1. H-FIRE waveform used in experiments. Each sample was exposed to 100 bursts of biphasic pulses, 2 µs negative and positive phase, with 5 µs interphase and 5 µs interpulse delay, with 25 pulses/burst, and 1 Hz burst repetition frequency. The graphs show one burst of biphasic pulses (left) and one biphasic pulse within the burst (right). The voltage set on the pulse generator was 200 V.

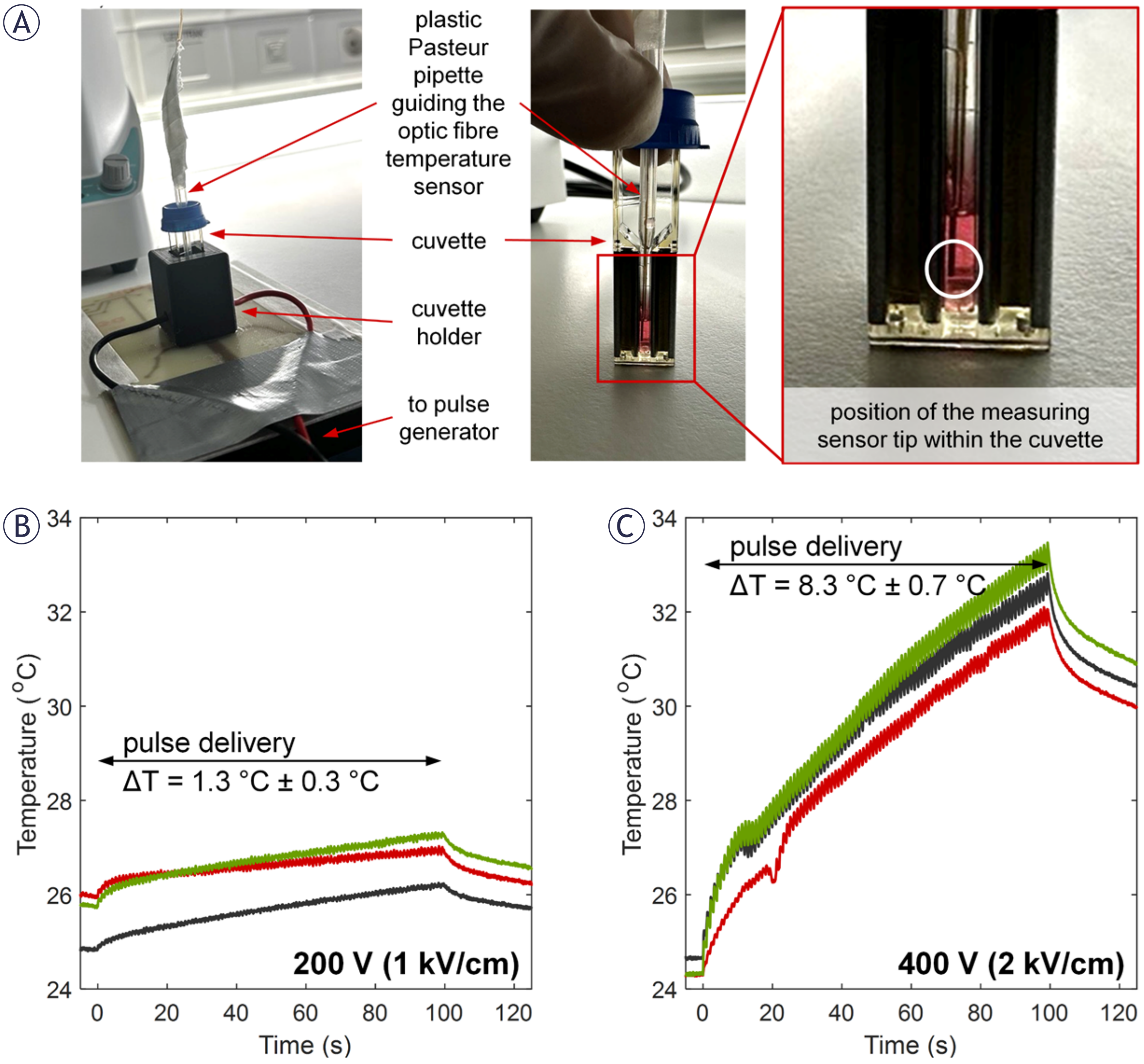

SUPPLEMENTARY FIGURE S2. Increase in sample temperature during delivery of the H-FIRE waveform. **(A)** Temperature measurements were performed using fibre optic sensor MPK-5 (OpSens Solutions, Quebec, Canada) during delivery of electric pulses at ambient temperature. The sensor was inserted into electroporation medium between the electrodes inside the cuvette. A plastic Pasteur pipette, cut at the top and bottom side, was used to guide the optic fibre and keep it in place during measurements. **(B)** Temperature recordings from 3 samples exposed to H-FIRE waveform with amplitude of 200 V **(C)** and 400 V. The ΔT shows the difference in temperature at the end and the beginning of pulse delivery (mean ± standard deviation of the three measurements).

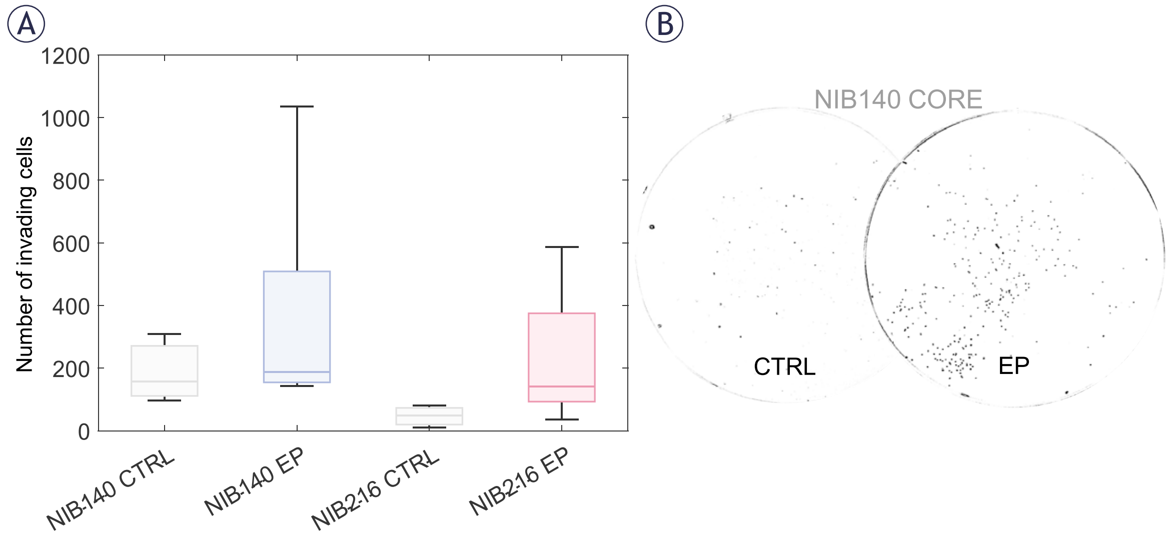

SUPPLEMENTARY FIGURE S3. Increased invasion of patient-derived GB cells following exposure to H-FIRE waveform resulting in electric field strength of 2 kV/cm. **(A)** Quantification of the number of invading cells in NIB140 and NIB216 CORE cell lines. Electroporated (EP) samples showed a trend toward increased invasion compared to untreated controls (CTRL); however, the differences were not statistically significant for neither NIB140 CORE (Mank-Whitney Rank test, p = 0.517) nor NIB216 CORE (Student's t-test; p = 0.159). Data are presented as box plots showing median, interquartile range, and full data range from three biological replicates (1–3 technical replicates per biological replicate). **(B)** Representative image of transwell inserts highlighting increased invasion following electroporation for NIB140 cell line.
